# Supplementary material for: Two-dimensional semiconducting SnP2Se6 with giant second-harmonic-generation for monolithic on-chip electronic-photonic integration
Source: Nat Commun. 2023 May 2;14:2521. doi: 10.1038/s41467-023-38131-2 (PMC10154306; doi:10.1038/s41467-023-38131-2)
Supplement: Supplementary file 1 — Supplementary Information [file 41467_2023_38131_MOESM1_ESM.pdf]

Supplementary Information for

Two-dimensional semiconducting SnP_2Se_6 with giant second-harmonic-generation for monolithic on-chip electronic-photonic integration

Cheng-Yi Zhu[1#], Zimeng Zhang[2#], Jing-Kai Qin[1*], Zi Wang[2], Cong Wang[3], Peng Miao[4], Yingjie Liu[2], Pei-Yu Huang[1], Yao Zhang[2], Ke Xu[2*], Liang Zhen[1,5], Yang Chai[3*] and Cheng-Yan Xu[1,5*]

[1] Sauvage Laboratory for Smart Materials, School of Materials Science and Engineering, Harbin Institute of Technology (Shenzhen), Shenzhen 518055, China

[2] Guangdong Provincial Key Laboratory of Semiconductor Optoelectronic Materials and Intelligent Photonic Systems, Harbin Institute of Technology, Shenzhen 518055, China

[3] Department of Applied Physics, The Hong Kong Polytechnic University, Hong Kong, China

[4] HORIBA Scientific, Shanghai 205335, China

[5] MOE Key Laboratory of Micro-Systems and Micro-Structures Manufacturing, Harbin Institute of Technology, Harbin 150080, China

[#] These authors contributed equally to this work

[*] E-mail addresses: jk.qin@hit.edu.cn; kxu@hit.edu.cn; ychai@polyu.edu.hk; cy_xu@hit.edu.cn

Table of Contents

1. Experimental setup of SCCVT and materials characterization
2. DFT calculations
3. Polarization-dependent SHG measurement of SnP_2Se_6
4. SHG measurement of $MoTe_2$ and susceptibility calculation
5. Fabrication and performance of SnP_2Se_6 field effect transistor
6. Optoelectronic properties of SnP_2Se_6 photodetector
7. Numerical simulations of the SHG process in SiN microring resonator
8. Optical measurement of device based on SnP_2Se_6/SiN hybrid structure
9. Investigation of SHG conversion efficiency with the thickness of the transferred SnP_2Se_6 on the SiN microring resonator
10. Fabrication of SiN microring resonator

1. Experimental setup for the SCCVT and materials characterization

The precursors including Sn powder, P powder and Se powder were sealed into a quartz tube with vacuum pressure less than 10 mPa, together with fluorophlogopite mica as the growth substate. A microreactor with confined space is formed by stacking freshly cleaved mica vertically, as shown in **Supplementary Figure 1**. The microreactor would help to decrease the concentration of precursors and contribute to the uniform growth of SnP_2Se_6.

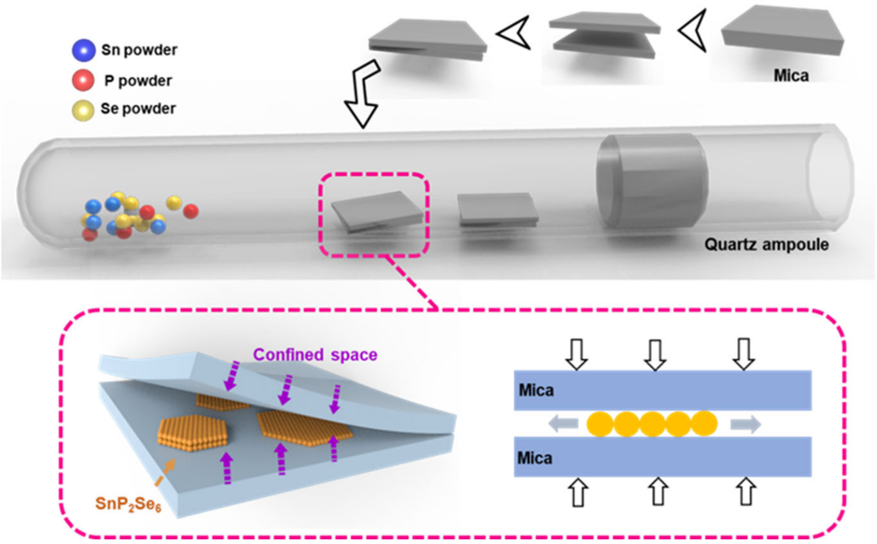

Supplementary Figure 1| Schematic of the self-limited epitaxy strategy to grow 2D SnP_2Se_6 atomic crystals.

Supplementary Figure 2a and **2b** shows the OM image and corresponding AFM height profile of a large-size single-crystalline SnP_2Se_6 nanosheet, the maximum lateral size can reach up to 1 millimeter with a thickness of about 18.5 nm. 2D SnP_2Se_6 nanosheets were obtained between two pieces of mica substates with confined space, in which the products are attached to the surface of mica. Therefore, the SnP_2Se_6

nanosheets usually exhibit clear terraces with stepped thickness when the microreactor was peeled off (**Supplementary Figure 2c**). As shown in **Supplementary Figure 2d**, the thickness of a terrace is about 0.7 nm, corresponding to the thickness of monolayers, confirming the layered structure of SnP_2Se_6.

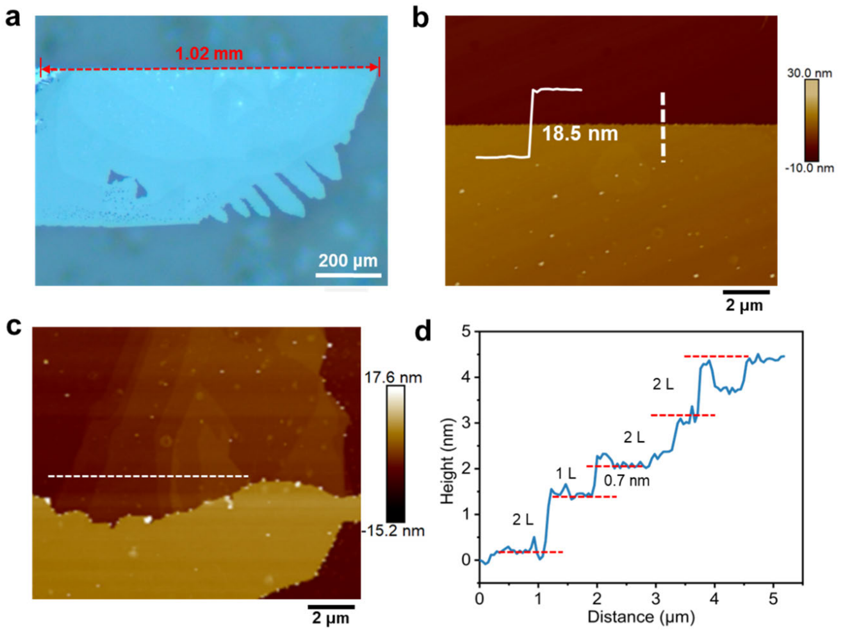

Supplementary Figure 2| a, OM image and **b,** height profile of a large-size single-crystalline SnP_2Se_6 nanosheet (the maximum lateral size can reach up to 1 millimeter) with a thickness of about 18.5 nm. **c,** AFM topography image and **d,** height profile of a SnP_2Se_6 nanosheet with different layer numbers.

The high-resolution X-ray photoelectron spectroscopy (XPS) spectrum was conducted on the as-grown samples on mica substate. As shown in **Supplementary Figure 3**, we can see that the oxidation state of Se^{2-} was clearly identified by the two characteristic peaks of Se $3d_{5/2}$ (~ 54.8 eV) and Se $3d_{3/2}$ (~ 55.9 eV), while the two peaks at 487.5 and 495.9 eV can be attributed to the Sn $3d_{5/2}$ and Sn $3d_{3/2}$ satellite,

respectively. In addition, another two specific peaks were also observed, including P $2p_{3/2}$ and P $2p_{1/2}$ at 132.6 and 134.8 eV. The results demonstrate the synthesis and ideal atomic stoichiometry of the SnP_2Se_6 nanosheets.

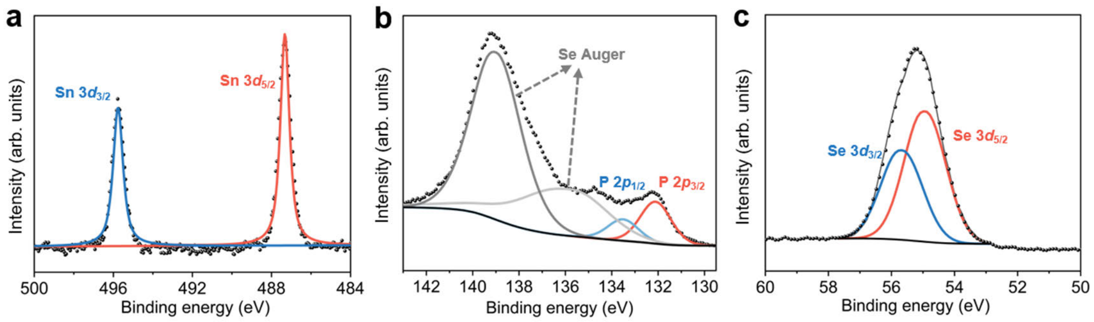

Supplementary Figure 3| High-resolution XPS spectra for **a,** Sn 3*d*, **b,** P 2*p* and **c,** Se 3*d* of SnP_2Se_6 nanosheets.

Angle-resolved polarized Raman spectroscopy (ARPRS) was performed on a thin SnP_2Se_6 nanosheet with thickness of about 15 nm. As shown in **Supplementary Figure 4a**, the sample exhibits typical hexagonal shape, and the angle of 0° is defined when the polarization direction of laser is along the angular point direction. **Supplementary Figure 4b** plots the Raman spectra of sample from 0 to 180° in a step of 7.5°, from which we can see that the intensity of Raman peaks would change periodically, exhibiting a characteristic 6-fold rotational symmetry. The polar figure plotted by extracting data belonging to P_3 exhibits 6-fold rotational symmetry (**Supplementary Figure 4c**), which is indicative of the isotropic vibrational characteristics of SnP_2Se_6 crystals.

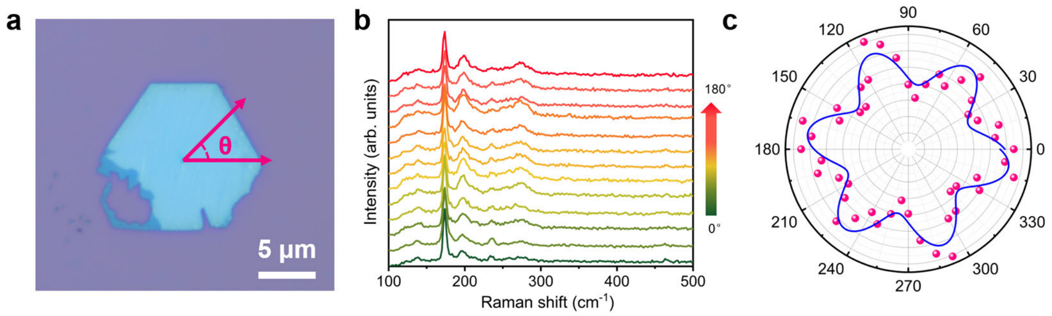

Supplementary Figure 4| a, Optical microscope image of SnP_2Se_6 nanosheet with the definition of polarization angle. **b,** The evolution of Raman spectrum with the angle from 0 to 180° and **c,** corresponding polar figures of peak intensity.

High-angle annular dark field scanning transmission electron microscope (HAADF-STEM) was used to characterize the SnP_2Se_6 nanosheets. Elemental energy-dispersive X-ray spectroscopy (EDS) measurements give the density of the elements (**Supplementary Figure 5**). The sample utilized for TEM characterization has a thickness of about 14 nm. After eliminating the influence of Cu from TEM grid, we can see that element including Sn, P and Se are clearly detected, together with perfect stoichiometric ratio around 1:2:6 for SnP_2Se_6. EDS mapping was also performed, from which we can see that all the elements are distributed uniformly across the sample, indicating the high crystalline quality of SnP_2Se_6.

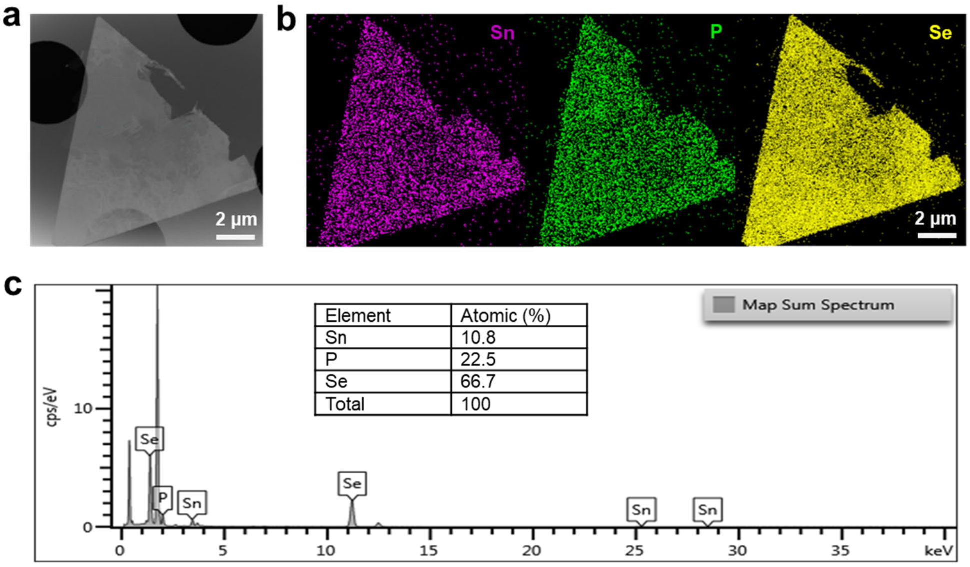

Supplementary Figure 5| a-b, Element distribution of SnP_2Se_6 obtained by EDS mapping. **c,** EDS spectrum of the sample.

2. DFT calculations of band structure

First-principles DFT calculations were carried out to predict the electric structure of SnP_2Se_6. The crystal structure of a monolayer SnP_2Se_6 crystal is shown in **Supplementary Figure 6a**. To prevent the unphysical interactions between layers, a vacuum space of 20 Å thick is used along the [001] direction. Since the exchange–correlation functional of the Perdew–Burke–Ernzerhof (PBE) generalized gradient approximations (GGA) generally underestimates the absolute bandgap energy of semiconductors, HSE06 was employed to better estimate the value of band gap (E_g). HSE06 yields E_g of the relaxed structure (1.74 eV) for monolayer SnP_2Se_6, close to the experimental value (1.90 eV). According to the corresponding partial density of state (**Supplementary Figure 6b**), we can see that Sn and Se atoms make the major contributions to the lowest conduction band, whereas the highest valence band is mainly constructed by Se atoms.

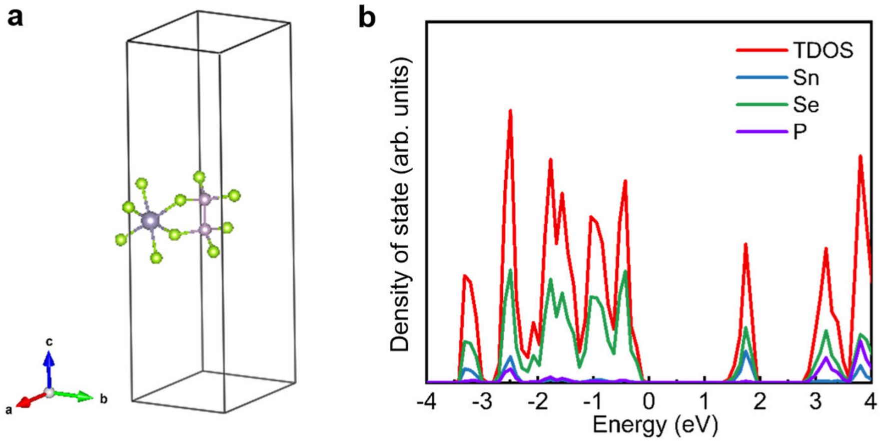

Supplementary Figure 6| a, The supercell of monolayer SnP_2Se_6 for DFT calculations. **b,** Calculated partial density of state (DOS) for SnP_2Se_6 monolayer.

3. Polarization-dependent SHG measurement of SnP_2Se_6

We carried out polarized SHG measurements to investigate the NLO properties of SnP_2Se_6. A femtosecond (fs) laser with a central wavelength of 1550 nm was linearly polarized and focused on the samples. The SHG intensity of samples were collected by rotating the sample with a step of 15° from 0° to 360°. The incident laser was initially polarized along the armchair axis of SnP_2Se_6.As shown in **Supplementary Figure 7**, the intensity changes periodically with the change of angle, and it can be fitted using the following formular[1]:

$$I = I_0 \sin^2 3\theta \quad (1)$$

where I is the SHG intensity at angle θ, and I_0 is the maximum SHG intensity.

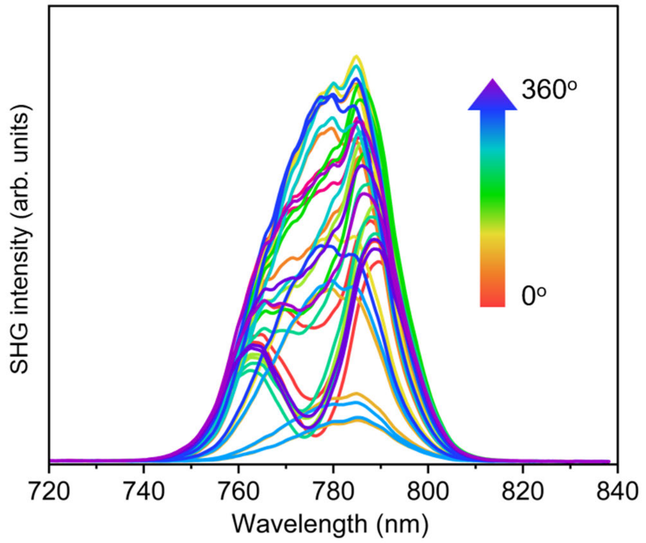

Supplementary Figure 7| Polarization-dependent SHG signals of SnP_2Se_6 nanosheet in parallel configuration under 1550 nm excitation wavelength.

The polarized SHG measurements also provide us a feasible approach to determine the crystalline quality of samples. **Supplementary Figure 8a** is an OM image of a

SnP_2Se_6 nanosheet with thickness of about 20 nm. As scanning the laser across the sample, we can get the SHG intensity mapping shown as **Supplementary Figure 8b**. The uniform color distribution indicates the sample is highly-crystallized in single crystal.

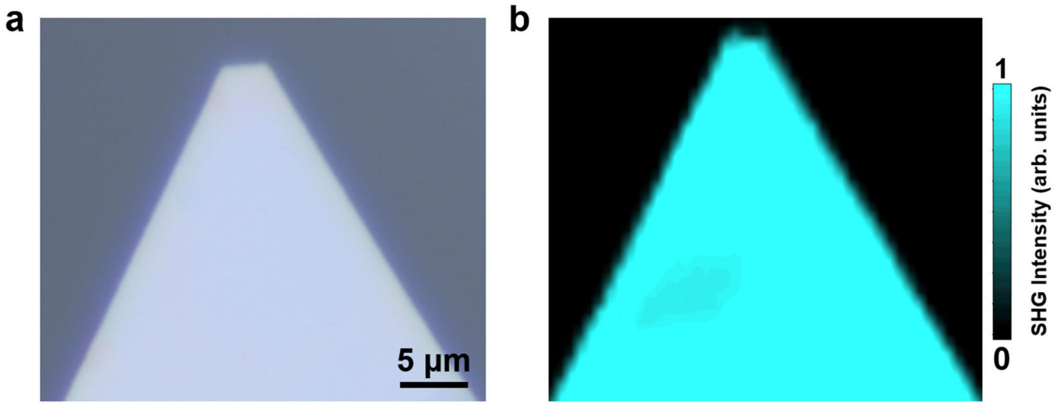

Supplementary Figure 8| a, OM image and **b,** related SHG intensity mapping of SnP_2Se_6 nanosheet under 1550 nm excitation wavelength.

4. SHG measurement of $MoTe_2$ and susceptibility calculation

Semiconducting TMDC monolayers are expected to exhibit strong SHG response. However, most of them deliver relatively low susceptibility at telecom wavelength (1310−1550 nm). Among of them, a highest susceptibility ($\chi^{(2)}$ ~2.5×10^{-9} mV^{-1}) was demonstrated for $MoTe_2$ monolayer, thus it is utilized as the counterpart for comparison. $MoTe_2$ nanosheets were obtained through a mechanical exfoliation process, and the OM image is shown as **Supplementary Figure 9c**. Similar to the case of SnP_2Se_6, the SHG intensity of $MoTe_2$ nanosheet also changes periodically with angle, showing six-fold characteristics (**Supplementary Figure 9b**). The measured SHG intensity for 35 nm-thick (~50 layer) SnP_2Se_6 is several orders of magnitude larger than 4 nm-thick (~5 layer) $MoTe_2$, as plotted in **Supplementary Figure 9d**. We can estimate susceptibility of SnP_2Se_6 using the following equations[2]:

$$P_{2\omega} = \frac{8\pi^2 d^2}{\varepsilon_0 c\lambda^2 A} \cdot \frac{\left[\chi^{(2)}\right]^2}{n_\omega^2 n_{2\omega}} \cdot P_\omega^2 \quad (2)$$

$$\chi^{(2)} = \frac{{\varepsilon_0}^{1/2} c^{1/2} \lambda A^{1/2}}{8^{1/2}\pi} \cdot \frac{1}{P_\omega} \cdot \frac{1}{d} \cdot P_{2\omega}^{1/2} \cdot n_\omega n_{2\omega}^{1/2} \quad (3)$$

where P_ω and $P_{2\omega}$ represent the excitation laser power and SHG power, respectively; d is the thickness of the sample; ε_0 and c are dielectric constant and the speed of light in vacuum, respectively; A is the area of incident laser spot; n_ω and $n_{2\omega}$ denote the linear refractive indices of the sample at the fundamental and SH frequencies, respectively.

As $P_{2\omega}$ of the SnP_2Se_6 sample cannot be measured directly, the second-order nonlinear susceptibility of SnP_2Se_6 ($\chi^{(2)}_{\mathrm{SnP2Se6}}$) can be measured by comparing with $MoTe_2$ in identical test conditions.

$$\chi^{(2)}_{\mathrm{SnP2Se6}} = \frac{I^{1/2}_{2\omega-\mathrm{SnP2Se6}}}{I^{1/2}_{2\omega-\mathrm{MoTe2}}} \cdot \frac{d_{\mathrm{MoTe2}}}{d_{\mathrm{SnP2Se6}}} \cdot \frac{n^{1/2}_{2\omega-\mathrm{SnP2Se6}}}{n^{1/2}_{2\omega-\mathrm{MoTe2}}} \cdot \frac{n_{\omega-\mathrm{SnP2Se6}}}{n_{\omega-\mathrm{MoTe2}}} \cdot \chi^{(2)}_{\mathrm{MoTe2}} \quad (4)$$

According to previous report, the second-order nonlinear susceptibility of $MoTe_2$ ($\chi^{(2)}_{\mathrm{MoTe2}}$) at 1560 nm excitation wavelength is about 2.5×10^{-9} m V^{-1}, we can estimate $\chi^{(2)}_{\mathrm{SnP2Se6}}$ is approach 1.32×10^{-9} m V^{-1} for SnP_2Se_6.

Particularly, the value of $\chi^{(2)}$ relies on accurate knowledge of several experimental parameters, such as frequency and duration of the excitation pulse, the shape and size of the focused fundamental spot at the sample, and the relation between the measured spectral counts and the actual SH power. Hence, the values should be viewed as an order of magnitude estimate for references[3].

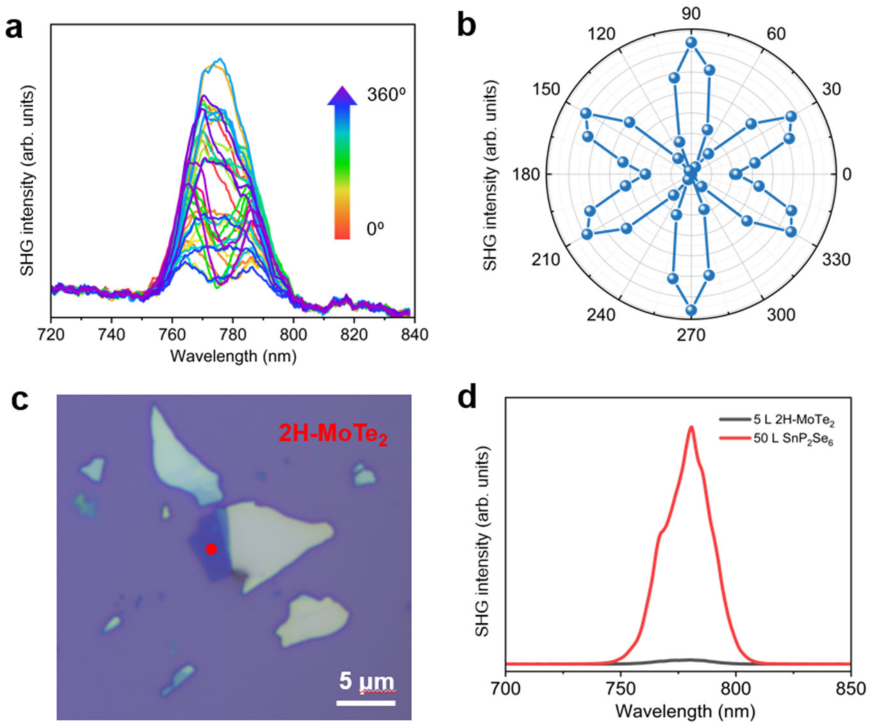

Supplementary Figure 9| a-b, Polarization-dependent SHG signals of $MoTe_2$ in parallel configuration under 1550 nm excitation wavelength. **c,** OM image of $MoTe_2$. **d,** SHG signal comparison of 5L-$MoTe_2$ and 50 L-SnP_2Se_6 obtained under identical conditions.

5. Fabrication and performance of SnP_2Se_6 field effect transistor

To investigate the electrical properties, field-effect transistors were fabricated after transferring SnP_2Se_6 nanosheets onto SiO_2/Si substrate with the assistance of PMMA. Ti/Au (10/50 nm) electrodes were patterned with standard photolithography process followed by electron beam evaporation. **Supplementary Figure 10a** and **10b** show the OM image of device and corresponding AFM height profile of a device, in which the channel length is 5 μm and thickness is about 10 nm. The switching characteristics was obtained in vacuum ($\sim 1\times10^{-4}$ mbar) at room temperature **(Supplementary Figure 10c** and **10d)**. The channel switched from the 'OFF' state to the 'ON' state as the gate voltage varied from −50 V to 50 V, showing a typical electron-dominated transport behavior. The Ti-SnP_2Se_6 interface demonstrates a linear output characteristic at different gate voltages, which is indicative of good Ohmic contact on the hole side of the gate doping. The carrier mobility could be extracted from the linear part of transfer curve using the expression:

$$\mu = L/W\times(1/C_g)\times dI/dV \quad (5)$$

where L, W, and C_g represent the FET channel length, width, and gate dielectric capacitance, respectively. The value of electron mobility is estimated to be 6.5 $cm^2\ V^{-1}\ s^{-1}$ at room temperature.

In addition, we have estimated the contact resistance of the FET device. The total resistance of a single field-effect transistor includes two parts: channel resistance and contact resistance ($R=R_{channel}+2R_{contact}$). We have constructed field effect transistors based on 16-nm-thick SnP_2Se_6 film with different channel lengths ranging from 2 to

8.7 μm (**Supplementary Figure 10e**), and each device has the same contact electrode to ensure the consistency of contact resistance. **Supplementary Figure 10f** exhibits the total resistance of different transistor in the on-state mode (V_g=40 V). According to the intercept value of the ordinate, we can estimate that the $R_{contact}$ of the device is about 24.5 kΩ·μm.

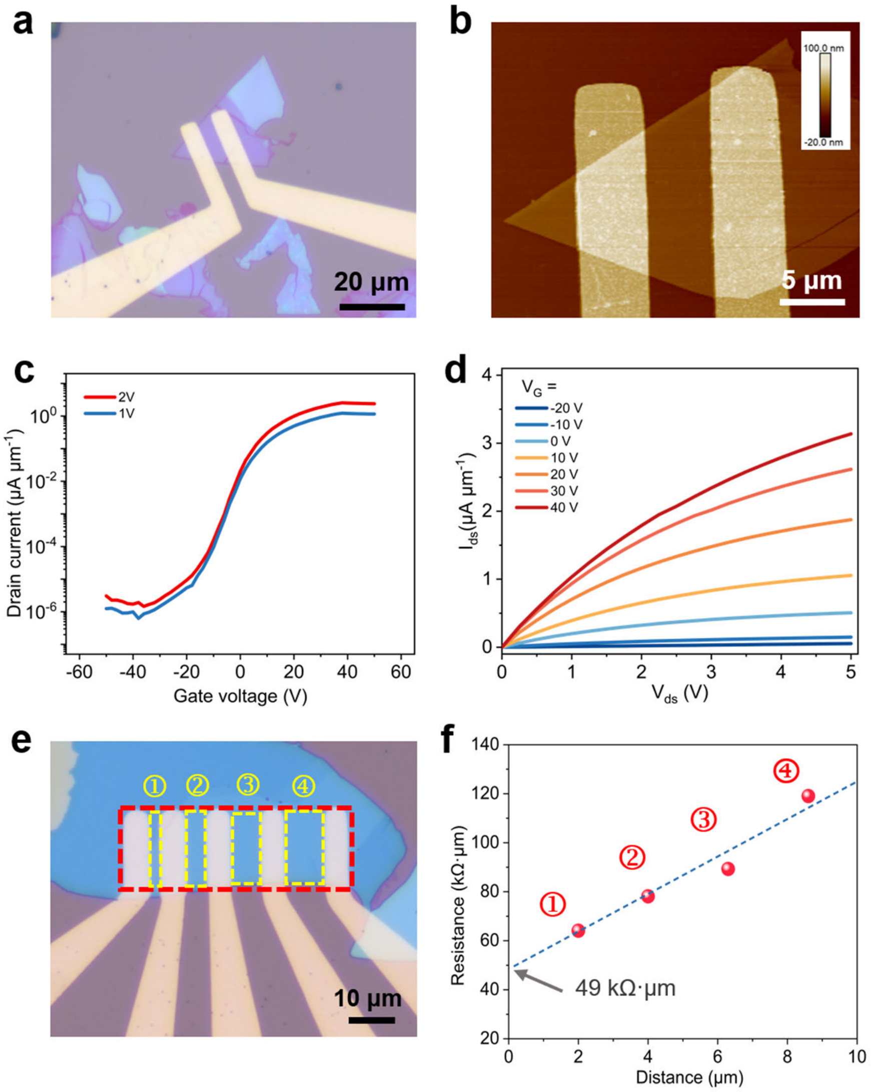

Supplementary Figure 10| a, OM and **b,** AFM topography images of SnP_2Se_6 FET device. **c,** Typical transfer curves of the SnP_2Se_6 FET (10 nm-thick). Channel length of the device is 5 μm. **d,** The output curves of device at different gate voltage. **e**, OM image and **f**, total resistance of the device with different channel lengths.

Thickness-dependent field-effect mobility and ON-OFF ratio were evaluated based on the data from more than forty devices. By extracting the data from the linear region of transfer characteristics, we can obtain an electron mobility of 15 $cm^2\ V^{-1}\ s^{-1}$ for a 20-nm-thick sample. Due to the enhanced gate electrostatic control in thin nanosheets, the ON/OFF ratio increases sharply from ~10^2 to ~10^5 as the nanosheet thickness decreases from 40 to 5.6 nm. On the contrary, the carrier mobility exhibits the opposite trend, ranging from 0.2 $cm^2\ V^{-1}\ s^{-1}$ to 10 $cm^2\ V^{-1}\ s^{-1}$ (**Supplementary Figure 11**). The interface scattering, which often occurs in FETs based on 2D ultrathin semiconductors, can be well utilized to explain the thickness-dependent characteristics[4,5].

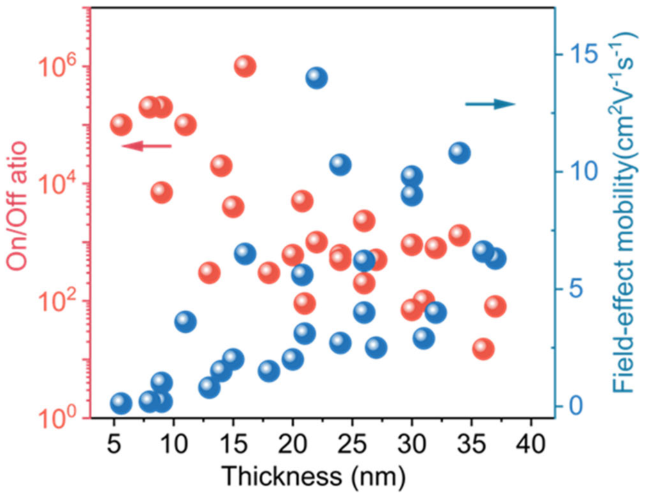

Supplementary Figure 11| Distributions of measured on/off current ratios and field-effect mobilities obtained from 30 typical SnP_2Se_6 FETs with different thicknesses at room temperature

As shown in **Supplementary Figure 12**, we measured the transfer curves of a 10-nm-thick SnP_2Se_6 field effect transistor with temperature ranging from 7 to 300 K. As the temperature decreases from 300 to 200 K, the measured mobility would increase slightly from 3 to 4.5 $cm^2\ V^{-1}\ s^{-1}$. With the further decreasing of temperature, the mobility would drop gradually, and a value of 0.6 $cm^2\ V^{-1}\ s^{-1}$ was estimated.

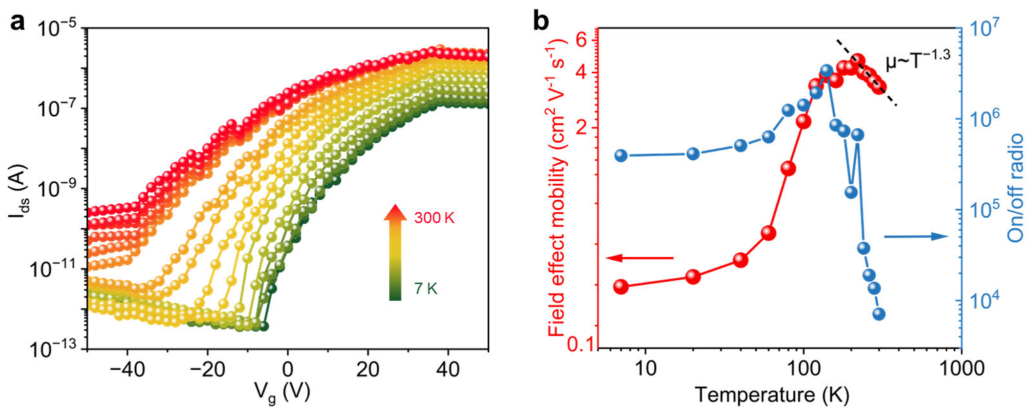

Supplementary Figure 12| a, Transfer curves of the SnP_2Se_6 FET (10 nm-thick) at different temperature ranging from 7 to 300 K. **b,** Temperature dependent field effect mobility and on/off radio obtained from **a.**

6. Optoelectronic properties of SnP_2Se_6 photodetector

For the wavelength-dependent photodetection measurement, a broadband laser-driven light with excitation wavelength ranging from 300 to 900 nm was utilized. The power intensity of light was calibrated and fixed at 2 mW cm^{-2} for the measurement. **Supplementary Figure 13** plots the *I-V* curves of 10-nm-thick phototransistor as exposed to different wavelengths of light, and the maximum photocurrent can be achieved with 700 nm illumination.

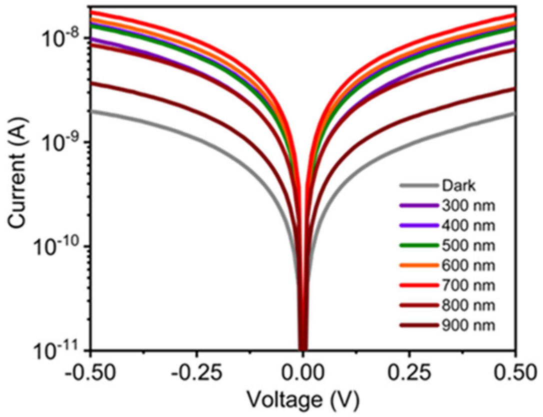

Supplementary Figure 13| *I-V* curves of device as a function of wavelength ranging from 300 to 1000 nm with laser power density of 20 mW cm^{-2}.

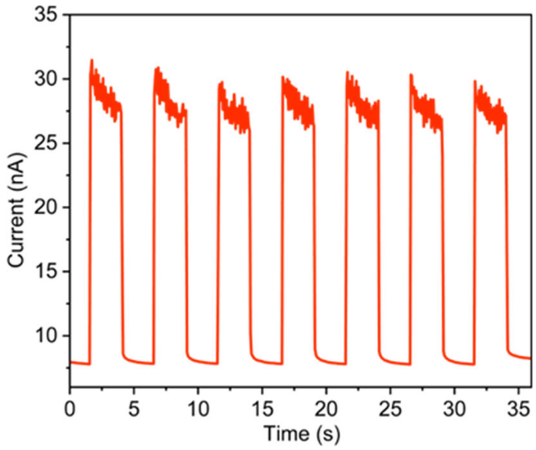

Supplementary Figure 14| Time-resolved photoresponse of the SnP_2Se_6 Photodetector at a bias voltage of 1 V under 700 nm illumination with an optical power density of 20 mW cm^{-2}.

The time-resolved photoresponse of device was investigated by manually turning the laser ON/OFF periodically with a time interval of 5 s at V_{bias}=1 V. As shown in **Supplementary Figure 14**, the periodically repeated photocurrent was revealed without degeneration even after seven cycles, indicating the good stability and reproducibility of the device.

7. Numerical simulations of the SHG process in SiN microring resonator

The microring resonator is fabricated on 300 nm-thick SiN slab with a diameter of 35 μm, and the coupling length is designed to be 7.5 μm. With the assistance of a 2D finite-difference eigenmode solver, we calculated the effective refractive indices of guiding modes in SiN microring resonator. The wavelength of fundamental pump laser is chosen to be 1550 nm. We studied the dependence of the effective refractive indices on the strip width of the microring (**Supplementary Figure 15**). Both effective refractive indices for fundamental pump laser (1560 nm) and SHG signals (780 nm) increase with width of microring. Equal effective refractive indices can be obtained at w = 1.24 μm, which means that the phase matching condition is satisfied for the SHG process at this strip width.

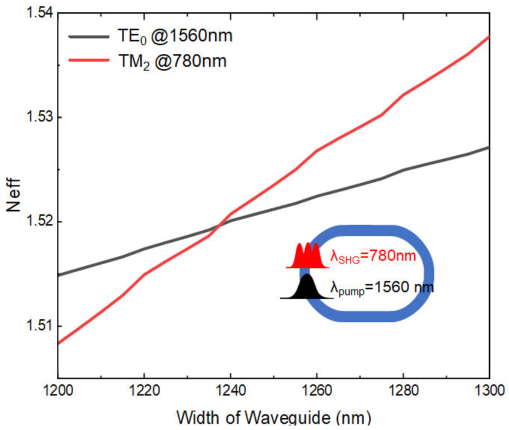

Supplementary Figure 15| Calculated effective refractive indices of resonance modes for the fundamental pump laser and SHG signals with the changing of strip width. Inset image depicts the resonance modes in microring.

The guiding mode profiles of fundamental pump laser and the SHG signals in SiN microring resonator are calculated. As shown in **Supplementary Figure 16**, to

minimize the transmission loss, TE_0@1560 nm (with the polarization parallel to the waveguide plane) is considered for the fundamental pump light in WG_{pump} and microring due to the waveguide bending and small radius of the SiN microring. After frequency conversion from 1560 to 780 nm, TM_2@780 nm (with the polarization normal to the waveguide plane) should be considered in microring to realize the maximum frequency conversion efficiency.

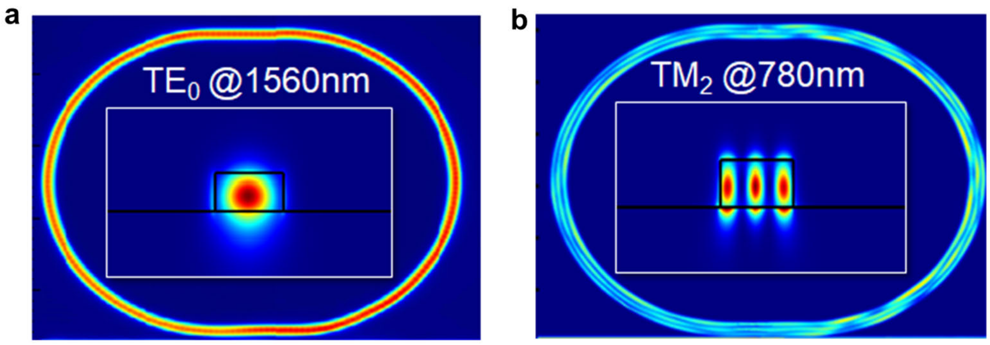

Supplementary Figure 16| a, Mode profiles of the fundamental pump TE_0 mode and **b,** SHG TM_2 mode in the SnP_2Se_6–SiN microring with a strip width of 1.23 μm.

To reduce the transmission loss, the TM_2 guiding mode in microring is finally converted to fundamental TM_0 mode in WG_{pump} through a mode converter, and the simulated conversion efficiency can reach up to 96% (**Supplementary Figure 17**).

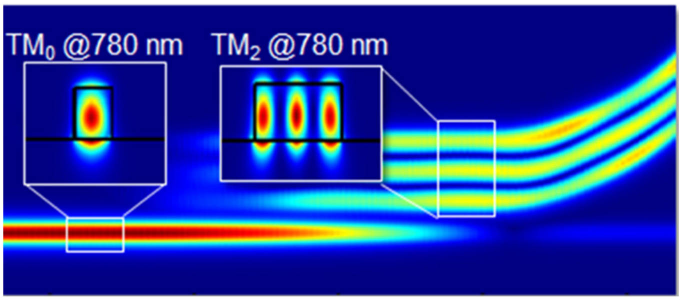

Supplementary Figure 17| The mode conversion from TM_2 in microring to TM_0 in sub-waveguide through a mode converter.

8. Optical measurement of device based on SnP_2Se_6/SiN hybrid structure

The testing device set-up consists of a tunable laser (Santec TSL-710) at 1560 nm, a polarization controller, a fiber-chip coupling stage and a benchtop power meter (MPM210). As shown in **Supplementary Figure 18**, the fundamental pump laser is coupled into the SiN waveguide through a polarization controller and a single-mode polarization-maintaining fiber, to ensure the incident mode as TE_0. The tilt angle is designed to be 10° with respect to the surface normal direction (see the inset picture of **Supplementary Figure 18**) to suppress the back-reflection. **Supplementary Figure 19** is the transmission spectra of fundamental pump laser, showing a strong emission peak with a center wavelength of about 1560 nm.

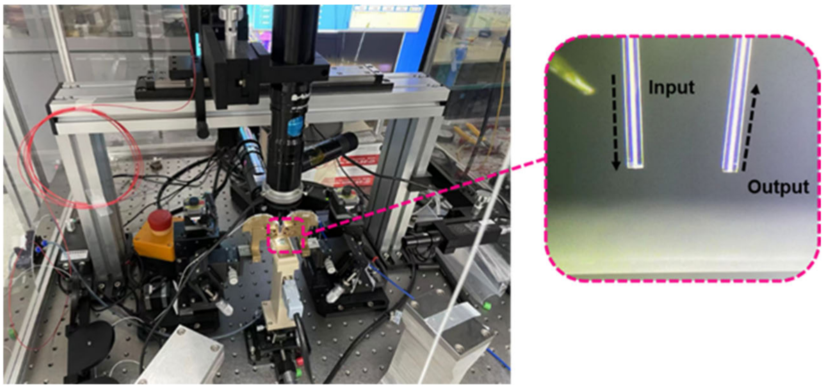

Supplementary Figure 18| Digital photograph of the device for on-chip waveguide testing, while the SHG excitation source is vertically coupled into the waveguide through a single-mode polarization-maintaining fiber with an inner diameter of 10 μm.

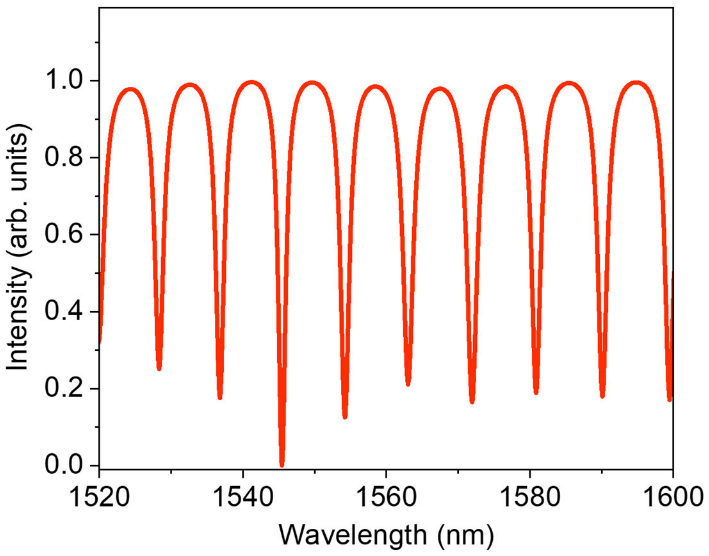

Supplementary Figure 19| Normalized transmission spectra of the SiN microring.

9. Investigation of SHG conversion efficiency with the thickness of the transferred SnP_2Se_6 on the SiN microring resonator

The SHG efficiency for a lossless waveguide without pump depletion is given by the following expression[6,7]:

$$\eta_{\mathrm{TE0-TM2}} = \frac{P_{\mathrm{SH}}}{P_{\mathrm{FF}}^2} \times 100\% = \xi_{\mathrm{NL}}^2 L^2 \frac{sin^2(\Delta\beta L/2)}{(\Delta\beta L/2)^2} \times 100\% \tag{6}$$

The Q value in the micro ring determines the overall length of propagation L, $\Delta\beta = 2\beta^{\mathrm{pump}} - \beta^{\mathrm{SHG}}$ represents the wave vector mismatch, and ξ_{NL} represents nonlinear overlap factor, which can be defined as:

$$\xi_{NL} = n^2 \chi^{(2)}_{\mathrm{SnP2Se6}}\, sin3\theta \left(\frac{8\pi^2}{\varepsilon_0 c \lambda_{\mathrm{FF}}^2 n_{\mathrm{SH}} n_{\mathrm{FF}}^2}\right)^{\frac{1}{2}} \left(\frac{\int_{\mathrm{SnP2Se6}} \left(E_{\mathrm{FF}}^2 x\right)^2 E_{\mathrm{SH}} y\, dxdz}{\left(\int_{\mathrm{all}} |\overrightarrow{E_{\mathrm{FF}}}| \mathrm{dxdz}\right)^2 \left(\int_{\mathrm{all}} |\overrightarrow{E_{\mathrm{SH}}}| dxdz\right)}\right) \tag{7}$$

In this formular, θ is the angle formed by the guided-mode wave vector and the armchair direction of the SnP_2Se_6 crystal. However, since our device is fabricated based on a micro ring, this term can be eliminated by integral. Considering this, the equation (7) can be reduced to:

$$\xi_{\mathrm{NL}} = n^2 \chi^{(2)}_{\mathrm{SnP2Se6}} \left(\frac{8\pi^2}{\varepsilon_0 c \lambda_{\mathrm{FF}}^2 n_{\mathrm{SH}} n_{\mathrm{FF}}^2}\right)^{\frac{1}{2}} \left(\frac{\int_{\mathrm{SnP2Se6}} \left(E_{\mathrm{FF}}^2 x\right)^2 E_{\mathrm{SH}} y\, dxdz}{\left(\int_{\mathrm{all}} |\overrightarrow{\boldsymbol{E_{FF}}}| \mathrm{dxdz}\right)^2 \left(\int_{\mathrm{all}} |\overrightarrow{\boldsymbol{E_{SH}}}| dxdz\right)}\right) \tag{8}$$

where $\chi^{(2)}_{\mathrm{SnP2Se6}}$ is the second-order nonlinear susceptibility of SnP_2Se_6, n is the layer of the material, λ_{FF}=1550 nm is the pump wavelength, n_{FF} and n_{SH} represents the effective refractive indices of the TE_0 mode at fundamental frequency and TM_2 mode at Second-harmonic frequency modes, respectively. ε_0 and c are the permittivity and light speed in vacuum. $\int_{\mathrm{SnP2Se6}}$ and $\int_{\mathrm{all}}$ denote two-dimensional integration over SnP_2Se_6 and all space, respectively. $E_{\mathrm{FF}}x$ is the x component of $E_{\mathrm{FF}}(x,z)$, the electric field of the fundamental mode TE_0, and $E_{\mathrm{SH}}y$ is the y component of $E_{\mathrm{SH}}(x,z)$, the

electric field of the second-harmonic mode TM_2. These electric field distribution data are obtained by Lumerical MODE solutions.

In fact, $n^2\chi^{(2)}_{SnP2Se6}$ reflects the SHG conversion intensity of the SnP_2Se_6 with different layers. From **Figure 3b**, we noted that the SHG intensity reveals a near-quadratic dependence when the layer is less than 7, while the light re-absorption would lead to the deviation from the quadratic relationship, even showing a downward trend in the range from 8 to 70 layers. Therefore, we can obtain the fact value of $n^2\chi^{(2)}_{SnP2Se6}$ (**Supplementary Figure 20**).

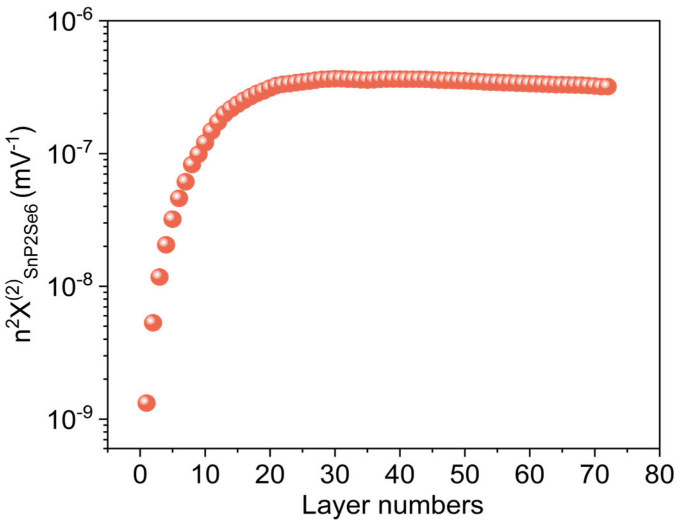

Supplementary Figure 20| The fact value of $n^2\chi^{(2)}_{SnP2Se6}$ with the layer numbers of SnP_2Se_6.

By substituting the data from **Supplementary Figure 20** into equation (8) and equation (6), we can theoretically calculate the SHG conversion efficiency. **Supplementary Figure 21** shows the theoretical variation trend of the SHG conversion efficiency with material thickness. Different material thicknesses would result in various propagation constants, electric field distribution of incident waves, and second harmonics for the situation of fixed propagation length and waveguide width, which is the primary cause for the difference in SHG conversion efficiency. Here, the waveguide

width was set to be 1.26 μm and the bending radius of the micro ring to 25 μm. According to **Supplementary Figure 21**, we can conclude that the number of material layers providing the optimal SHG conversion efficiency is around 64 in this device configuration. It's important to note that this value is highly relative to the fixed device. Once the size parameter of the micro ring changes, the optimal conversion layer of the material will also change.

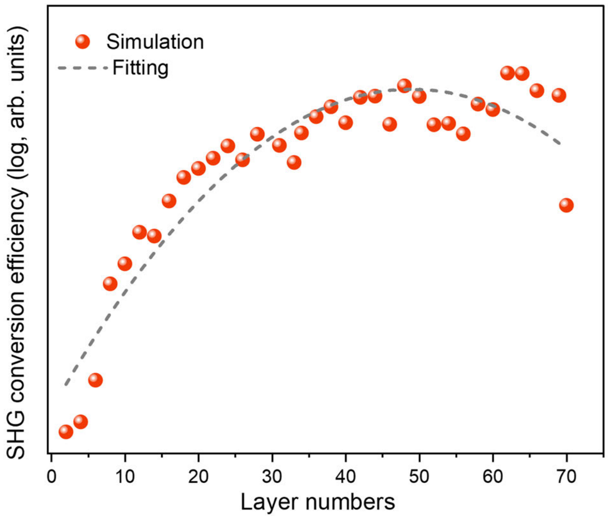

Supplementary Figure 21| The variation of SHG conversion efficiency with the thickness of the transferred SnP_2Se_6 on the micro ring device, as measured by logarithmic scale.

To further prove the above conclusion, we have supplemented the experimental data of SHG efficiency in micro ring structures covered with different thicknesses of SnP_2Se_6 (**Supplementary Figure 22**). The experimental results show that the SHG conversion efficiency increases with the thickness from 12 to 21 nm, while it drops slightly for the 36 nm-thick sample. The result is consistent with the theoretical analysis trend shown in **Supplementary Figure 21**.

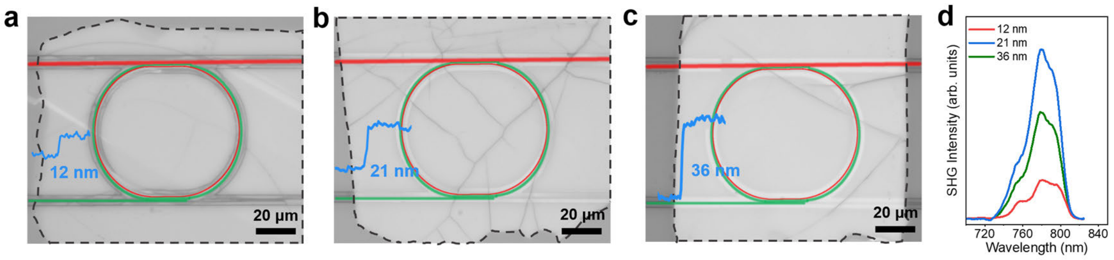

Supplementary Figure 22| OM image of SnP_2Se_6 of different thicknesses transferred onto micro ring. **d,** SHG efficiency measured at different thicknesses of SnP_2Se_6.

In summary, our device has preliminarily verified the feasibility of integration of frequency doubling conversion and photoelectric detection, but the specific performance indexes need to be further improved. Waveguide structure, *Q* value of resonant ring, sample thickness and other factors will have a great impact on the performance of the device, which will be the focus of our research in the future.

10. Fabrication of SiN microring resonator

Supplementary Figure 23 depicts the fabrication process of a SiN microring resonator. It is fabricated on a 300 nm-thick SiN slab, which are deposited on Si substrate coated with a 3 μm-thick buried oxide layer. The widths of two coupling bus waveguides are different, which are designed to accomplish the coupling in of the fundamental pump laser (at 1560 nm) and coupling out of the SHG signals (at 780 nm). A 200 nm-thick SiO_2 cladding is deposited by the electron beam evaporation above SiN slab, and the devices are patterned by the electron beam lithography using positive photoresist. The SiO_2 layer is then fully etched to a depth of 200 nm, and the patterned layer of SiO_2 is utilized as the hard mask for the following SiN etching. Both two steps of etching are carried out with the assistance of inductively coupled plasma dry etching.

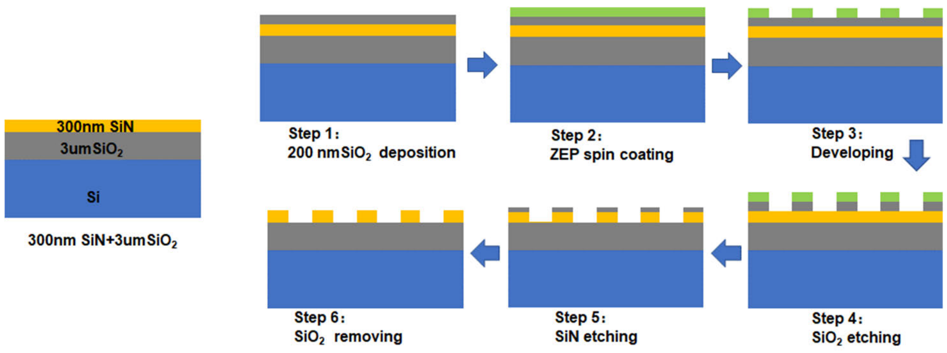

Supplementary Figure 23| Fabrication process of the SiN microring resonator.

Supplementary references

1 Malard, L. *et al.* Observation of intense second harmonic generation from MoS_2 atomic crystals. *Phys. Rev. B* **87**, 201401 (2013).

2 Shi, J. *et al.* 3R MoS_2 with broken inversion symmetry: a promising ultrathin nonlinear optical device. *Adv. Mater.* **29**, 1701486 (2017).

3 Kumar, N. *et al.* Second harmonic microscopy of monolayer MoS_2. *Phys. Rev. B* **87**, 161403 (2013).

4 Lin, M. *et al.* Thickness-dependent charge transport in few-layer MoS_2 field-effect transistors. *Nanotechnology* **27**, 165203 (2016).

5 Feng, W. *et al.* Back gated multilayer InSe transistors with enhanced carrier mobilities via the suppression of carrier scattering from a dielectric interface. *Adv. Mater.* **26**, 6587-6593 (2014).

6 Chen, H. *et al.* Enhanced second-harmonic generation from two-dimensional $MoSe_2$ on a silicon waveguide. *Light: Sci. Appl.* **6**, e17060 (2017).

7 Luo, R., *et al.* Highly tunable efficient second-harmonic generation in a lithium niobate nanophotonic waveguide. *Optica* **5**, 1006-1011 (2018).
